# Severe epidemic myalgia with an elevated level of serum interleukin-6 caused by human parechovirus type 3: a case report and brief review of the literature

**DOI:** 10.1186/s12879-018-3284-5

**Published:** 2018-08-07

**Authors:** Kiwamu Nakamura, Kyoichi Saito, Yasuka Hara, Tetsuji Aoyagi, Kadzuhiro Kitakawa, Yoshinobu Abe, Hiromu Takemura, Fumihito Ikeda, Mitsuo Kaku, Keiji Kanemitsu

**Affiliations:** 1 Department of Infection Control, Fukushima Medical University, 1 Hikarigaoka, Fukushima, 960-1295 Japan; 2 Ikeda Memorial Hospital, Sukagawa, Japan; 3 Department of Infection Control and Laboratory Diagnostics, Tohoku University Graduate School of Medicine, Sendai, Japan; 4 Department of Microbiology, Fukushima Prefectural Institute of Public Health, Fukushima, Japan; 5 Department of Microbiology, St. Marianna University School of Medicine, Kawasaki, Japan

**Keywords:** Human parechovirus type 3, Epidemic myalgia, Orchiodynia, IL-6

## Abstract

**Background:**

Human parechovirus type 3 (HPeV-3) is known to cause cold-like symptoms, diarrhea, or severe infections such as sepsis in infants and children. In adults, HPeV-3 infection is rarely diagnosed because the symptoms are generally mild and self-limiting; however, this infection has been linked to epidemic myalgia, regardless of the presence of underlying diseases, immunosuppression, or sex.

**Case presentation:**

We describe an adult case of severe systemic myalgia and orchiodynia after infection with HPeV-3, which was transmitted from the child of the patient. Interleukin-6 (IL-6) level was found to be elevated in the patient’s serum.

**Conclusion:**

Severe myalgia associated with HPeV-3 infection is potentially caused by an elevated serum level of IL-6.

## Background

Human parechovirus type 3 (HPeV-3) was first reported in 2004 [1] and has since been identified to cause cold-like symptoms, diarrhea, or severe infections such as meningitis and sepsis-like disease in neonates [2]. However, HPeV-3 is rarely diagnosed in adults because the symptoms are generally mild and conventional clinical laboratory diagnostic tests are unavailable; accordingly, it is difficult to determine the etiology. Adult cases of epidemic myalgia associated with HPeV-3 were initially reported in Japan in 2012 [3], and other adult cases of myalgia due to HPeV-3 have since been reported [4–6]. In addition, myalgia was also reported to occur in children [7]. Interestingly, all reported adult cases of epidemic myalgia due to HPeV-3 have occurred only in Japan, despite the ubiquity of HPeV-3 in Europe, Asia, and the USA [8–10]. We herein describe an adult case of severe systemic myalgia and orchiodynia after infection with HPeV-3, which was transmitted from the child of the patient.

## Case presentation

A previously healthy 32-year-old man, presented to the outpatient department of our institution with a 3-day history of high fever, sore throat, and mild diarrhea in early September 2016. His chief complaints were severe myalgia in both sides of his cervical and trunk muscles (around the pectoralis major, rectus abdominis, and trapezius areas), in addition to muscles of the upper and lower extremities (both proximal and distal), and orchiodynia. Additionally, he complained of inadequate sleep due to severe leg pain that led him to fear being unable to rise from bed after lying down. He therefore stood by his bed throughout the night.

On physical examination, the patient’s height was 171 cm and body weight was 67 kg (body mass index = 22.9). There was no paresis or muscle tenderness noted, and all deep-tendon reflexes were normal. His pain did not extend to the facial, hand, foot, or joint regions. No tenderness was observed in the testes, despite the complaint of orchiodynia. Rectal examination did not indicate prostatitis. He was fully conscious, and no paresis, speech disturbance, or skin eruptions were observed. The differential diagnoses initially included periodic paralysis, myasthenia gravis, adult-onset Still’s disease, fibromyalgia, and chronic fatigue syndrome.

An antigen-based rapid diagnostic test detecting both influenza virus A and B yielded a negative result. His white blood cell count was 3700/mm^3^, serum C-reactive protein (CRP) level was 1.41 mg/dL (normal range: < 0.2 mg/dL), serum creatine phosphokinase (CK) level was 48 U/L (normal range: 60–230 U/L), and serum myoglobin level was 63.1 ng/mL (normal range: 20.3–92.3 ng/mL). All liver and thyroid function tests, electrolytes, and serum ferritin level were within normal limits. Two sets of blood cultures both yielded negative findings. Circulating anti-nuclear, anti-acetylcholine receptor, and anti-neutrophil cytoplasmic antibodies were not detected.

At the time of case presentation, the patient’s wife had just delivered a daughter and was temporarily staying at her parents’ house. The patient and his wife also had a 3-year-old son, with whom the patient stayed at their own home following his wife’s parturition. He worked in an office and sent his son to a nursery school during the daytime working hours. Five days before the patient’s initial visit to our hospital, his son developed a fever and mild throat pain, and several infants at the nursery school also developed mild flu-like symptoms and mild diarrhea that improved over 2–3 days. To rule out the possibility of enterovirus infection, serum antibodies were tested for coxsackievirus (type A2, A4, A5, A6, B2, B4, B5, B6) and echovirus (type 13, 30) at the initial visit and 2 weeks later with the neutralization test method. Significant antibody titer changes between acute and convalescent phases were not detected and a serological diagnosis was not established for these enteroviruses. Because adult HPeV-3 infection may occur shortly after an epidemic of pediatric infection, nested polymerase chain reaction (PCR)-based detection tests for HPeV-3 were performed, and the HPeV-3 types were identified by sequencing the VP3/VP1 junction in PCR products amplified directly from the specimens [11]. We collected blood samples from the patient at the first visit and 2 weeks later, and obtained stool samples from all family members several days later. We also collected specimens from the patient’s parents because the severity of his myalgia restricted his ability to move freely in his house, and he required assistance from his parents in caring for his son and himself.

**Fig. 1:**
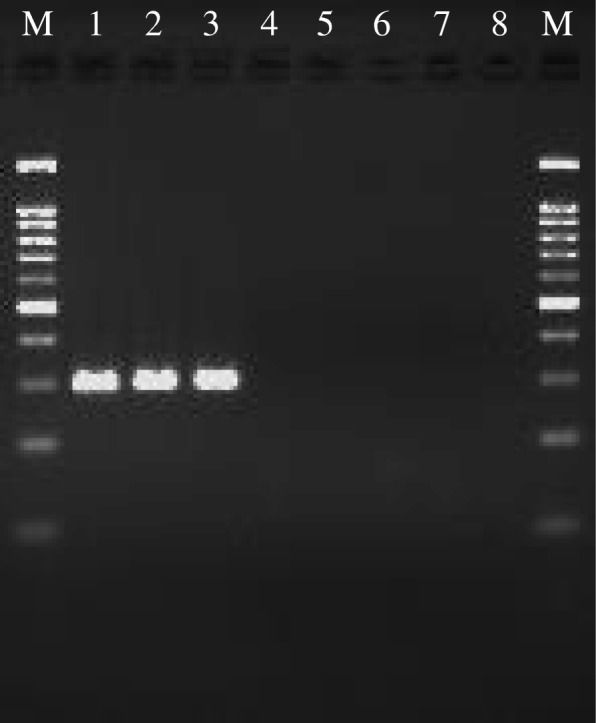
Nested polymerase chain reaction for human parechovirus-3 in specimens from the patient and his family. The target human parechovirus-3 polymerase chain reaction product length was 300 base pairs. Lanes: 1, serum of the patient at the first visit; 2, stool of the patient; 3, stool of his son; 4, stool of his wife; 5, stool of his neonatal daughter; 6, stool of the patient’s father; 7, stool of the patient’s mother; 8, serum of the patient at 2 weeks after the first visit; M, 100-base pair size marker

HPeV-3 was detected in the patient’s initial blood and stool samples and his son’s stool sample (Fig. 1); viable HPeV-3 viruses were isolated from stool specimens using a cell culture method. We confirmed that the sequences of the PCR-amplified regions were identical in specimens from the patient and his son, indicating that the son had transmitted HPeV-3 to the patient. The results from the mother and newborn daughter, who had not stayed with the patient and his son during that time, were negative. The results from the patient’s parents were also negative.

He was finally diagnosed with severe epidemic myalgia caused by HPeV-3 infection. Without specific treatment, his severe myalgia, fever, and orchiodynia gradually improved. After a week, he had no difficulty in moving his whole body, and no sequelae remained. Additionally, we measured serum cytokine levels at the initial visit and 2 weeks later. Tumor necrosis factor-α (TNF-α), interleukin-6 (IL-6), IL-10, and IL-12 levels were measured by enzyme-linked immunosorbent assays. A cut-off value for each assay was as follows: TNF-α and IL-10, below 2 pg/mL; IL-6 and IL-12, below 4 pg/mL. Although the values of his TNF-α, IL-10, and IL-12 levels were all below each cutoff value, his IL-6 level was elevated to 63.6 pg/mL at the initial visit. Two weeks later, his IL-6 level had decreased to almost half (34.9 pg/mL), but remained higher than normal.

## Discussion and conclusions

HPeV-3 is thought to circulate among infants in nursery schools, as well as in households. However, most related cases of myalgia occur in adults and are thought to have been transmitted from young children [12]. In previous reports, most adult patients with epidemic myalgia due to HPeV-3 infection are aged between 20 to 40 years [3–6]. While the overall seropositivity rate (cut-off value of greater than 8×) for HPeV-3 was 79.4% in residents of Yamagata prefecture (next to Fukushima prefecture, where the case patient lived), the necessary antibody titer for preventing HPeV-3 infection (neutralizing [NT] antibody titer equal to or higher than 32×) decreases in adults as they age [13]. This might suggest that adults who do not have sufficient titers of NT antibodies may develop HPeV-3 infection after occasional contact with infected children, and a few individuals may have further complication, such as with epidemic myalgia.

Previously, Mizuta et al. reported 22 cases involving epidemic myalgia in adults (average age: 37 years [range: 23–66 years]) [3]. All but one patient had muscular weakness, and 18 of 22 (82%) presented with high fever. In those cases, severe myalgia mainly occurred in the proximal upper and/or lower limbs, and laterality was generally not recognized. Furthermore, Mizuta et al. reported elevated CRP levels in 86% of patients, although the values were relatively low (average: 1.95 [0.1–6.8] mg/dL). Elevated CK and myoglobin levels occurred in 55% (average: 443 [43–1598] mg/dL) and 73% (average: 101 [18–253] mg/dL) of patients, respectively, indicating that CK and myoglobin are not always elevated in patients with epidemic myalgia, regardless of severity. Other symptoms reported by Mizuta and colleagues included pharyngeal pain and orchiodynia, a specific symptom of epidemic myalgia with an unknown mechanism that was reported by 27% of male patients, as well as by our patient. In another report, Tanaka et al. used computed tomography to detect swollen testis and peritesticular effusion in a patient [4]. Yamakawa and Mizuta et al. also reported 17 confirmed epidemic myalgia adult cases (average patient age: 36.8 years [range: 21–50 years], 14 males and 3 females) in 2017 [7]. All patients had myalgia and muscular weakness; 14 patients (82%) presented with high fever, 8 patients (47%) exhibited upper respiratory symptoms, and 4 (24%) had gastroenteritis. Scrotal pain occurred in 4 of 14 male patients (29%). In these cases, all but one showed an elevated level of CK (average value: 1017 [61–6155] IU/L, normal value; 55–175 IU/L). Mild leukocytopenia occurred in 7 of 17 patients (average value: 4391 [2730–5610] cells/mm^3^, normal value; 3900–9000 cells/mm^3^). Increases in CRP value were mild (average value: 0.771 [0.21–5.138] mg/dL, normal value; 0.000–0.300 mg/dL) [6]. Interestingly, the levels of CK and CRP did not increase proportionally in these patients. The CRP level of the patient who had the highest CK level (6155 IU/L, patient number 5) was 0.342 mg/dL. On the other hand, the CK level of the patient who had the highest CRP value (5.138 mg/dL, patient number 14) was only slightly elevated (248 IU/L) [7]. In our case, despite the severe myalgia of the patient, which prevented him from laying down on the bed throughout the night, his CK level was normal and his CRP elevation was relatively low. There is the possibility that we did not detect CK elevation of the case patient due to the time points of our blood tests (initial visit, 2 days later, and 2 weeks later). However, systemic myositis including all extremities and trunk muscles is usually accompanied by a remarkable elevation of CK; additional mechanisms inducing systemic myalgia after HPeV-3 infection other than myositis are suggested in this patient.

In addition, many issues regarding epidemic myalgia due to HPeV-3 remain unclear. First, we have not yet determined why adult epidemic myalgia due to HPeV-3 has only been reported in Japan, despite the passage of several years since the first report. We might attribute this phenomenon to a racial variation, difference in the clinical testing system, or recognizability of this disease amongst general physicians (i.e., not pediatricians). Second, it remains unknown why only HPeV-3 causes severe epidemic myalgia in adults, as similar cases attributed to HPeV-1 or HPeV-2 have not yet been reported.

Compared to HPeV-3, influenza virus and enterovirus more commonly cause myalgia and myositis. Epidemic pleurodynia (also known as Bornholm disease) is generally caused by coxsackievirus type B, and less frequently by echovirus and coxsackievirus type A. This disease is characterized by fever and paroxysmal spasms of the chest and abdominal muscles. Most cases occur during localized summer outbreaks amongst adolescents and adults [14, 15]. In addition, severe rhabdomyolysis and renal failure can be induced by various viruses, including influenza viruses A and B [16]. In this case, however, the time frame in which our patient visited our institution was not in the local endemic influenza season (typically from late November to next March), and the antigen-based rapid influenza test result was negative. Serological assays for the tested coxsackievirus type A and B, or echovirus between the acute and convalescent phases did not indicate the acute infection.

Because the epidemiological evidence indicates that not all adults infected with HPeV-3 develop epidemic myalgia, we hypothesized that severe myalgia may be associated with an increase in cytokine production. As such, we measured the cytokine levels in serum samples from our patient at the initial visit and 2 weeks later. Among the cytokines measured, IL-6 level was exclusively elevated. IL-6 is a known inflammatory cytokine that causes pain by inducing prostaglandin E2 production. When administered to humans, IL-6 can cause side effects including fever, headache, and myalgia [17]. IL-6 has been correlated with the development of symptoms including myalgia caused by influenza virus infection [18]. In recent research, coxsackievirus type B4 was found to induce production of IL-6 together with interferon-α and TNF-α from human mononuclear cells [19]. Considering these reports and the current case, elevated levels of acute inflammatory cytokines may play a role in myalgia or myositis caused by viral infections. Although further studies are required to elucidate the pathogenesis of severe epidemic myalgia after HPeV-3 infection, the elevated level of IL-6 in the case patient’s serum may correlate with the development of this disease.

This is the first report to demonstrate increased IL-6 levels in an adult patient with epidemic myalgia due to HPeV-3 infection. Further virologic, genetic, epidemiologic, and pathologic studies are needed to verify our hypothesis regarding the relationship between elevated IL-6 levels and myalgia severity in adult patients with HPeV-3 infection.

## Abbreviations

CK: Creatine phosphokinase
CRP: C-reactive protein
HPeV-3: Human parechovirus type 3
IL: Interleukin
PCR: Polymerase chain reaction
TNF-α: Τumor necrosis factor-α
